# Efficacy of Brazilian Propolis Supplementation for Japanese Lactating Women for Atopic Sensitization and Nonspecific Symptoms in Their Offspring: A Randomized, Double-Blind, Placebo-Controlled Trial

**DOI:** 10.1155/2019/8647205

**Published:** 2019-09-11

**Authors:** Gen Igarashi, Takaaki Segawa, Naoe Akiyama, Tamon Nishino, Takeru Ito, Hiroshi Tachimoto, Mitsuyoshi Urashima

**Affiliations:** ^1^Division of Molecular Epidemiology, The Jikei University School of Medicine, Minato-ku, Tokyo 105-8461, Japan; ^2^Pfizer Japan Inc., Shibuya-ku, Tokyo 151-8589, Japan; ^3^Segawa Clinic, Mishima City, Shizuoka 411-0039, Japan; ^4^Department of Pediatrics, Fuji City General Hospital, Fuji City, Shizuoka 417-8567, Japan; ^5^Kapibara Kids Clinic, Minato-ku, Tokyo 108-0072, Japan; ^6^Alpaca Kids ENT Clinic, Toshima-ku, Tokyo 171-0052, Japan; ^7^Department of Pediatrics, Atsugi City Hospital, Atsugi, Kanagawa 243-8588, Japan; ^8^Department of Pediatrics, The Jikei University School of Medicine, Minato-ku, Tokyo 105-8461, Japan

## Abstract

Propolis is a natural product collected from several plants by honeybees and mixed with beeswax and salivary enzymes. In animal models, propolis suppressed IgE-mediated allergies. However, there is no clinical evidence that propolis prevents human atopic sensitization, to the best of our knowledge. Therefore, a randomized, double-blind, placebo-controlled trial was conducted to assess whether propolis supplementation for lactating women increases or decreases the level of total IgE and antigen-specific IgE in the serum of their offspring (i.e., atopic sensitization) at the time of their first birthday. In addition, whether propolis supplementation improves or worsens nonspecific symptoms (e.g., eczema) in the lactating women and their offspring was also investigated. This trial is registered with UMIN000020794. Eligible pairs of mothers and their offspring (*n*=80) were randomized to two groups: propolis (*n*=40) and placebo (*n*=40). Participants were evaluated every month, and 31 (78%) of the propolis group and 23 (58%) of the placebo group underwent blood tests at the first birthday of the offspring. Total IgE ≥ 10 U_A_/ml was seen in 26 (84%) infants whose mothers were given propolis, which was not significantly different from the 19 (86%) given placebo (*P*=0.80). Total IgE as a continuous variable was not significantly different between the propolis and placebo groups (*P*=0.70). Antigen-specific IgE levels for mites, egg white, cow's milk, and wheat, as both dichotomous and continuous variables, were not significantly different between the two groups. Both in mothers and their offspring, there were no significant differences in the subjective improvements of nonspecific symptoms between the two groups. Except for one mother who had transient and mild nausea, none of the other mothers or their offspring developed severe adverse events during the follow-up period. In conclusion, compared with placebo, Brazilian propolis supplementation did not influence the risk of atopic sensitization in infants and neither did it improve nor worsen nonspecific symptoms in either mothers or their infants.

## 1. Introduction

Propolis, so-called bee glue, is a natural resinous product that honeybees collect from various plants and mix with varying amounts of beeswax and salivary enzymes. Bees use propolis on their hives as protection against predators and microorganisms, to repair damage, as a thermal isolator, and to build aseptic locales to prevent microbial infection of larvae [1, 2]. In fact, propolis has been reported to show anti-infection, anti-inflammatory, antioxidant, immunomodulatory, and antitumor activities *in vitro* and/or *in vivo*, and it has a long history as a folk medicine [1, 3, 4]. Moreover, animal model studies suggested that propolis could suppress IgE-mediated allergies, e.g., asthma [5], food allergy [6, 7], and allergic rhinitis [8], by inhibiting degranulation of basophils [9] through NF*κ*B [10, 11]. Propolis includes various bioactive compounds such as phenolic compounds and vitamins that may provide health benefits [12–14]. However, to the best of our knowledge, there is no clinical evidence supporting these results in the animal models.

There is a possibility that IgE-mediated allergies are increasing globally. As an example, atopic dermatitis (AD) is one of the representative IgE-mediated allergies. AD is a chronic, inflammatory skin disorder characterized by disruption of epidermal barrier function as an aberrant immune response to antigens. Current therapies focus on symptom management by restoring epidermal barrier function with emollients and reducing inflammation [15, 16]. Emollients and topical corticosteroids are effective for most patients and are the agents most commonly prescribed by experienced dermatologists. According to a systematic review by Reynolds et al., supplementation with vitamins E and D has the most robust evidence for AD symptom management and probiotics may play a role in the prevention of infantile AD. Fatty acids such as docosahexaenoic acid, sea buckthorn oil, and hempseed oil also have preliminary evidence supporting their use as supplements to decrease AD severity [16].

The prevalence of childhood AD has increased dramatically in the world over the past few years. The data from the National Health Interview Survey, a US population-based household survey, indicate that the prevalence of childhood AD has increased steadily from approximately 8% in 1997 to more than 12% in 2010 and 2011, but it may have plateaued in 2012 and 2013. A systematic review of 69 studies examining international trends in AD between 1990 and 2010 demonstrated childhood AD prevalence rates greater than 20% in some developed nations, with increasing rates of AD in Africa, eastern Asia, western Europe, and parts of northern Europe [17]. In Japan, according to the results of the nationwide survey by the Ministry of Health, Labour and Welfare (MHLW), 12.8% of 4-month-old, 9.8% of 18-month-old, 13.2% of 3-year-old, 11.8% of 7-year-old, and 10.6% of 12-year-old children were assessed as having AD at health examinations [18]. AD has subjective symptoms and can be a heavy burden, especially for children. Children and parents have a strong desire to find a solution. This strong desire is applicable to asthma, allergy rhinitis, and other IgE-related allergic diseases as well.

Thus, a randomized, double-blind, placebo-controlled trial was conducted to assess whether propolis supplementation for lactating women increases or decreases the level of total IgE and antigen-specific IgE in the serum of their offspring (i.e., atopic sensitization) sampled at the time of their first birthday. In addition, whether propolis supplementation improves or worsens nonspecific symptoms (e.g., eczema) in the lactating women and their offspring was also investigated.

## 2. Materials and Methods

### 2.1. Trial Design

This was a multicenter, double-blind, placebo-controlled trial to assess the safety of propolis supplementation for lactating mothers and their infants and to evaluate its efficacy for atopic sensitization by measuring IgE at the first birthday of the infants. The trial protocol was approved by the Ethics Committee of the Jikei University School of Medicine, and the institutional review board of Fuji City General Hospital, as well as of Atsugi City General Hospital. Written, informed consent was obtained from each participating mother. An external data and safety monitoring committee was set up for this study. This trial is registered with the UMIN Clinical Trials Registry (UMIN000020794).

### 2.2. Participants

The inclusion criteria were lactating mothers having atopic symptoms and their infants having eczema (or dry skin) around the face or neck between 2 and 8 months of age. The exclusion criteria were as follows: (1) maternal age less than 20 years; (2) infants with baseline disease other than atopic eczema; (3) mothers having food allergy to propolis and its ingredients; and (4) exclusive cow's milk feeding. All clinical data were collected at each collaborating medical institution and monitored at the Division of Molecular Epidemiology, Jikei University School of Medicine.

### 2.3. Randomization and Blinding

Computer-generated and centrally administered randomization used permuted blocks of 4. Participants were randomized in a 1 : 1 ratio without stratification. With the exception of MU and the staff of the data monitoring center at the Jikei University School of Medicine, who prepared the package of propolis or placebo according to the randomization assignment, all other collaborating pediatricians and participants were blinded to group assignment.

### 2.4. Intervention

The enrolled lactating mothers were randomly assigned to receive either the propolis 300® supplement including Brazilian green propolis (3 soft capsules/day) or the placebo (3 soft capsules/day), and they were asked to take the trial supplements from the day of enrollment when their offspring were between 2 and 8 months of age until their first birthday. Both supplements were supplied by Yamada Bee Farm Co., Ltd., Okayama, Japan. The quantity of propolis extract was 100 mg/capsule. The propolis was replaced with safflower oil in the placebo capsule. Both propolis and placebo capsules included perilla oil, wheat germ oil, lecithin, glycerin fatty acid ester, yellow bees wax, gelatin, glycerin, polysaccharide thickener, potassium chloride, and starch.

### 2.5. Outcomes

The primary outcome was atopic sensitization, i.e., serum levels of total IgE and antigen-specific IgE (mites, egg white, cow's milk, and wheat), with blood sampled at the first birthday, i.e., 12 months of age, outsourced for measuring with ImmunoCAP Specific IgE® (Thermo Fisher Diagnostics K.K., Minato-ku, Tokyo, Japan) to SRL Inc. (Hachioji-city, Tokyo, Japan) and compared as both continuous and dichotomous variables (total IgE ≥ 10 IU/ml and antigen-specific IgE (mites, egg white, cow's milk, and wheat) ≥0.35 U_A_/ml) between the propolis and placebo groups.

The secondary outcomes were improvement of nonspecific symptoms, e.g., eczema, in mothers and their offspring, evaluated subjectively at the first birthday.

Safety outcomes were nausea/vomiting and allergic reactions that disappeared soon after stopping the supplements. Severe adverse events were defined as those that required hospitalization.

### 2.6. Follow-Up

The participants were examined as outpatients every month.

### 2.7. Sample Size

The primary outcome, i.e., total IgE ≥ 10 IU/ml, was estimated to be 6% and 30% in the propolis and placebo groups, respectively, with a type I error (two-sided) of 5% and a power of 80%, assuming a 6% loss to follow-up. It was estimated that 100 participants divided into a 1 : 1 ratio would be sufficient to detect this difference. The *P* value for significance at the interim analyses, planned twice, at reaching 50 and 75 participating infants who had their first birthday, was set at <0.001 according to Peto's stopping boundaries [19].

### 2.8. Statistical Analysis

All participants who underwent randomization were included in this analysis. Outcomes were assessed according to the randomization group even when the intervention was not adhered to so-called intention-to-treat (ITT) analysis. Serum levels of IgE were compared using the Mann-Whitney test. Risk ratios (RRs) and 95% confidence intervals (CIs) were used to evaluate the dichotomous outcomes. The analyses were not corrected for multiple comparisons. *P* values <0.05 were considered significant. Stata 14.0 (StataCorp LP, College Station, TX) was used for all analyses.

## 3. Results

### 3.1. Study Population

**Figure 1:**
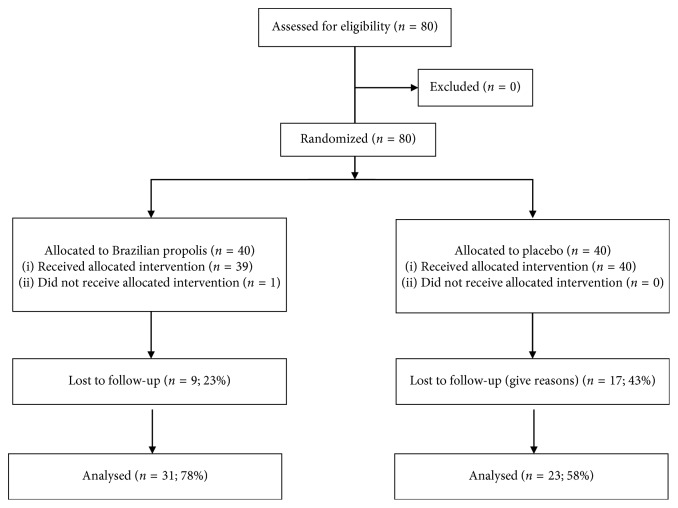
Trial profile.

Enrollment was stopped at 80, before the planned number of 100, because the interim analyses showed no trend in outcomes.  Figure 1 shows the flow diagram for the 80 participants who were randomly assigned to receive propolis supplements (*n*=40 : 50%) or placebo (*n*=40 : 50%) between June 2015 and September 2018 and followed up until January 2019. More than 90% of participants adhered to taking the supplements during the follow-up period. However, 23% of the propolis group and 43% of the placebo group were lost to follow-up before the first birthday of the infants although there was no significant difference (*P*=0.10).

The median (IQR) and maximum follow-up periods were 4 (3 to 5) and 8 months in the propolis group, respectively, and 4 (3 to 5) and 8 months in the placebo group, respectively.  Table 1 shows the characteristics of the participants. As infants' factors, the median age at randomization was 3.8 months in both groups. All factors were similar between the propolis group and the placebo group.

### 3.2. Effects of Propolis Supplementation on Total IgE at the First Birthday of the Infants

**Figure 2:**
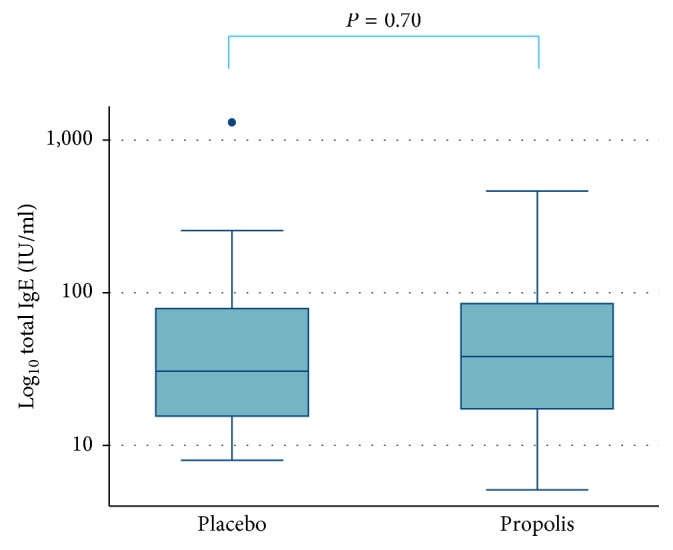
Box plots of total IgE levels (IU/ml) at the first birthday compared between the propolis and placebo groups. *P* values were calculated using the Mann–Whitney test. *Y*-axis shows common logarithm-transformed IgE levels.

Total IgE ≥ 10 IU/ml was found in 26 (84%) infants whose mothers were given propolis and in 19 (86%) of those given placebo (Table 2). Total IgE as a continuous variable was not significantly different between the two groups (Figure 2). Although median total IgE was higher though not significantly, in the propolis group than in the placebo group, its maximum value was 464 IU/mL in the propolis group, which was lower than the value of 1310 IU/mL in the placebo group (Table 2).

### 3.3. Effects of Propolis Supplementation on Antigen-Specific IgE Levels at the First Birthday

**Figure 3:**
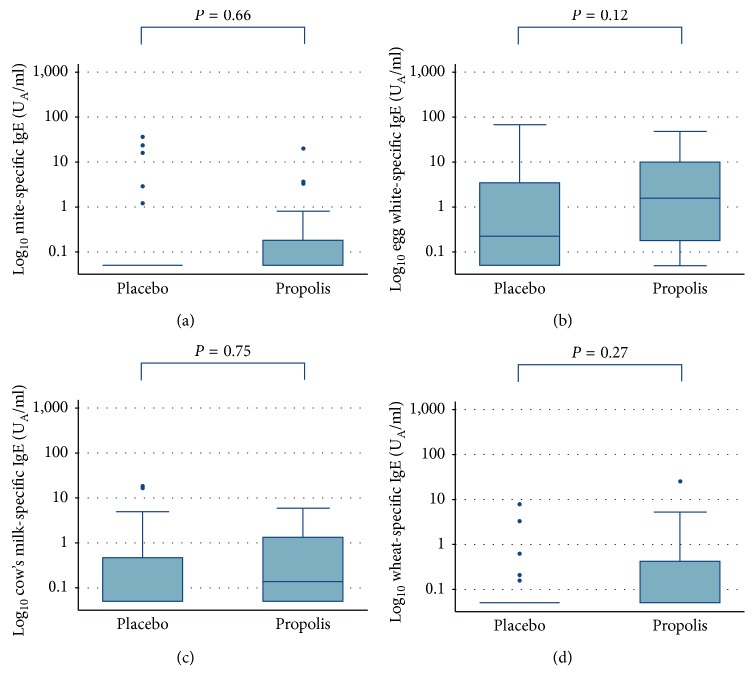
Box plots of antigen-specific IgE levels (U_A_/ml) at the first birthday compared between the propolis and placebo groups: mite-specific IgE (a); egg white-specific IgE (b); cow's milk-specific IgE (c); wheat-specific IgE (d). *P* values were calculated with the Mann–Whitney test. *Y* axes show common logarithm-transformed IgE levels.

Antigen-specific IgE (mites, egg white, cow's milk, and wheat) ≥0.35 U_A_/ml levels as dichotomous (Table 2) and continuous variables (Figure 3) was compared between the propolis and placebo groups. There were no significant differences. Although the median antigen-specific IgE levels tended to be higher though not significantly, in the propolis group, maximum values tended to be higher in the propolis group than in the placebo group, except for wheat-specific IgE (Table 2).

### 3.4. Improvement or Worsening of Nonspecific Symptoms

Subjective improvements of nonspecific symptoms in mothers and infants are shown in Table 3. Both in the mothers and infants, there were no significant differences in the subjective improvements of nonspecific symptoms between the propolis group and the placebo group. Withdrawal from usage of steroid ointment was observed in 3 (33%) of the placebo group, but in none (0%) of the propolis group, which was not significantly different (*P*=0.09). On the contrary, frequencies of worsening nonspecific symptoms were also not significantly different between the groups.

### 3.5. Adverse Events

One mother had nausea, which was improved one hour after she stopped taking the supplements.

## 4. Discussion

In pairs of mothers and their offspring at risk for atopic sensitization, propolis supplementation, compared with placebo, neither decreased nor increased atopic sensitization in the offspring, and neither improved nor worsened subjective symptoms, e.g., eczema, either in lactating women or their offspring. Regarding safety assessment, a mild adverse event of nausea was observed in one woman in the propolis group. Her symptoms improved one hour after stopping supplementation. It is well known that propolis has a unique smell that originates from its botanical ingredients and bee secretions. Some people do not feel comfortable taking propolis because of this smell. The nausea in the present study might have been caused by this smell. Moreover, nausea is a known adverse event caused by propolis that is mild and reversible. The present study showed that propolis did not appear to be associated with frequent or severe adverse events in lactating mothers and their offspring.

The strength of this trial was its study design, i.e., double-blind, randomized placebo-controlled trial, which enabled the evaluation of causal relationships of the subjective and nonspecific symptoms, e.g., eczema, as well as withdrawal from the steroid ointment. Propolis neither improved nor worsened these symptoms in the lactating mothers and their offspring, suggesting that lactating mothers may take propolis safely. To the best of our knowledge, there have been no randomized, double-blind clinical trials confirming the effects of propolis in lactating mothers and their offspring. Norizoe et al. conducted a randomized, double-blind clinical trial that showed that maternal supplementation with vitamin D increased food allergies in offspring who were breast-fed [20]. Using the same trial model, the present study suggested that lactating mothers could safely take propolis. These are meaningful positive findings for lactating mothers because they can take beneficial health supplements without anxiety for both them and their offspring.

As described in the Introduction section, animal models have suggested the potential efficacy of propolis for preventing and/or improving atopic conditions [5–11]. However, this trial did not show an effect of propolis on atopic sensitization. This negative result may suggest several possibilities: (1) Brazilian green propolis may not have any efficacy to improve atopic sensitization in humans; (2) the results of animal models may not apply to humans; (3) the sample size may have been too small to detect significant differences; (4) one-third of the participants were lost to follow-up, which may have resulted in a bias toward the null hypothesis; (5) the dose of propolis was too low to alter levels of IgE in the infants; (6) the trial supplements were started at a median age of 3.8 months, but the neonatal period may be a better time to start; (7) the follow-up period should be longer, such as by the 2^nd^ birthday; and (8) the ingredients of Brazilian green propolis may differ from those of other types of propolis.

There have been many reports suggesting the health benefits of Brazilian propolis, such as anti-infection, anti-inflammatory, antioxidant, immunomodulatory, and antitumor activities *in vitro* and *in vivo*. There are also some reports suggesting its efficacy in humans. Mujica et al. conducted a placebo-controlled human study that suggested positive effects on oxidative status and increased HDL-C, which is well known to reduce the risk of cardiovascular disease [21]. El-Sharkawy et al. suggested that propolis might improve periodontal therapy outcomes in people with type 2 diabetes mellitus and chronic periodontitis by showing significant improvements of glycemic control in a randomized, clinical trial (RCT) [22]. Pina et al. showed that propolis was noninferior to miconazole in older adults having denture stomatitis in a multicenter, randomized, open-label trial [23]. Although evidence in humans is still limited, the above reports suggest the possibility of health benefits in humans.

The approach of a randomized trial of a supplement in humans is meaningful. There are many randomized trials with drugs as standard approaches. However, generally, trials with supplements are limited because of funding issues. It is valuable to assess the efficacy of a traditional supplement shown in animals by a randomized trial in humans. In particular, subjective symptoms or improvement should be clarified by a randomized design without bias. If its efficacy is confirmed, it would suggest a significant health benefit that could be accessed broadly and easily. A group at the University of Miami Miller School of Medicine suggested the effect of a hydrolyzed polysaccharide supplement from rice bran on immunomodulatory biomarkers in adults with nonalcoholic fatty liver disease [24, 25]. A Korean research group suggested that acute grape seed extract can be an effective way to decrease cellular membrane damage after excessive exercise [26]. A meta-analysis of 3 RCTs found that vitamin D supplementation was significantly associated with lower total cancer mortality although each individual trial did not show it [27–30]. Our laboratory also tried to show that vitamin D improved *relapse-free* survival at 5 years in patients with digestive tract cancers in an RCT. The primary result was null but suggested that it was effective only in a subgroup of patients with middle (20–40 ng/mL) serum 25-hydroxyvitamin D levels at baseline [31]. These functional foods and supplements are easily obtained in daily life. Therefore, the reports improved our understanding of them.

The present study did not show a significant benefit of Brazilian green propolis on atopic sensitization in lactating mothers and their offspring. Although liver and kidney functions were not monitored, the findings suggested that there are no safety concerns for either the mothers or their offspring. However, Brazilian green propolis may contribute to health improvement of lactating mothers due to various benefits suggested in other trials. A further study with a larger number of subjects and earlier administration of supplement is needed to show the efficacy of propolis for preventing atopic sensitization.

## 5. Conclusions

In conclusion, compared with placebo, Brazilian propolis supplementation did not influence the risk of atopic sensitization in infants, and neither did it improve nor worsen nonspecific symptoms in either mothers or their infants.

## Figures and Tables

**Table 1:** Patients' characteristics^a^.

	Propolis, *n*=40	Placebo, *n*=40
*Medical institution*, *n (%)*		
Fuji City General Hospital	9 (22.5)	7 (17.5)
Atsugi City Hospital	1 (2.5)	3 (7.5)
Segawa Clinic	10 (25.0)	9 (22.5)
Kapibara & Alpaca Kids Clinic	20 (50.0)	21 (52.5)

*Perinatal factor*		
Gestational weeks		
Median (IQR)	39 (38–40)	39 (38–40)
Caesarean section, *n* (%)	3 (8)	4 (11)

*Infant factor*		
Months of age at randomization		
Median (IQR)	3.8 (2.9–5.5)	3.8 (2.9–5.5)
Birthweight (g)		
Mean (SD)	3074 (295)	3063 (406)

*Maternal factor*		
Maternal age (years)		
Mean (SD)	33.8 (5.2)	32.5 (7.0)
Maternal body mass index (kg/m^2^)		
Mean (SD)	21.0 (2.5)	20.8 (2.3)

*Maternal current atopic disease*, *n (%)*		
Asthma	3 (7.9)	1 (2.6)
Atopic dermatitis	6 (16)	6 (16)
Food allergy	6 (16)	4 (11)
Allergic rhinitis	19 (50)	13 (34)
Pollen allergy	26 (68)	25 (66)

*Maternal past atopic disease*, *n (%)*		
Asthma	6 (17)	4 (12)
Atopic dermatitis	11 (31)	5 (15)
Food allergy	3 (8.6)	2 (5.9)
Allergic rhinitis	8 (23)	3 (8.8)
Pollen allergy	7 (20)	4 (12)

*Paternal current atopic disease*, *n (%)*		
Asthma	2 (5.3)	2 (5.3)
Atopic dermatitis	3 (7.9)	5 (13)
Food allergy	2 (5.3)	4 (11)
Allergic rhinitis	7 (18)	8 (21)
Pollen allergy	21 (55)	15 (39)

*Paternal past atopic disease*, *n (%)*		
Asthma	4 (12)	5 (14)
Atopic dermatitis	8 (24)	5 (14)
Food allergy	3 (9.1)	2 (5.7)
Allergic rhinitis	2 (6.1)	2 (5.7)
Pollen allergy	7 (20)	4 (12)

^a^Due to rounding, total percentages are not always 100.

**Table 2:** Effects of propolis on atopic sensitization.

	Propolis	Placebo	Risk ratio	95% CI (upper–lower)	*P* value
*Total IgE*					
≥10 IU/mL *n* (%)	26 (84)	19 (86)	0.97	0.77–1.22	0.80
Median (25–75%)	38.3 (17.4–85.4)	30.75 (15.6–78.9)			
Range	5.1–464	8.0–1310			

*Mite-specific IgE*					
≥0.35 IU/mL	6 (21)	5 (22)	0.99	0.34–2.82	0.98
Median (25–75%) (U_A_/mL)	0.05 (0.05–0.185)	0.05 (0.05–0.05)			
Range (U_A_/mL)	0.05–19.9	0.05–36.2			

*Egg white-specific IgE*					
≥0.35 IU/mL	22 (71)	11 (48)	1.48	0.34–2.82	0.08
Median (25–75%) (U_A_/mL)	1.62 (0.18–10.0)	0.23 (0.05–3.41)			
Range (U_A_/mL)	0.05–47.2	0.05–67.2			

*Cow's milk-specific IgE*					
≥0.35 IU/mL	9 (29)	8 (38)	0.76	0.35–1.65	0.49
Median (25–75%) (U_A_/mL)	0.14 (0.05–0.98)	0.05 (0.05–0.48)			
Range (U_A_/mL)	0.05–5.99	0.05–18.3			

*Wheat-specific IgE*					
≥0.35 IU/mL	8 (26)	3 (14)	1.89	0.56–6.34	0.28
Median (25–75%) (U_A_/mL)	0.05 (0.05–0.43)	0.05 (0.05–0.05)			
Range (U_A_/mL)	0.05–25.3	0.05–7.88			

**Table 3:** Effects of propolis on improvement or worsening of nonspecific symptoms.

	Propolis, *n* (%)	Placebo, *n* (%)	Risk ratio	95% CI (upper–lower)	*P* value
*Improvement*					
Mother	4 (14)	3 (13)	1.06	0.26–4.26	0.94
Infants	11 (38)	9 (39)	0.97	0.49–1.93	0.93

*Steroid ointment withdrawal*					
Infants	0 (0)	3 (33)	0	—	0.09

*Worsening*					
Mother	3 (10)	1 (4.3)	2.30	0.26–20.7	0.44
Infants	2 (6.7)	1 (4.3)	1.53	0.15–15.9	0.72

## Data Availability

The EXCEL data used to support the findings of this study were supplied by Mitsuyoshi Urashima under license and so cannot be made freely available. Requests for access to these data should be made to Mitsuyoshi Urashima, E-mail: urashima@jikei.ac.jp.
